# Antifibrotic Effect of a Novel Selective 11β-HSD2 Inhibitor (WZ51) in a rat Model of Myocardial Fibrosis

**DOI:** 10.3389/fphar.2021.629818

**Published:** 2021-03-22

**Authors:** Fei Zhuang, Qin Ge, Jianchang Qian, Zhe Wang, Yaoyao Dong, Mengchun Chen, Xiaodan Zhang, Wei Sun

**Affiliations:** ^1^ Department of Pharmacy, The Second Affiliated Hospital and Yuying Children's Hospital of Wenzhou Medical University, Wenzhou, China; ^2^ Department of Endocrinology, Ningbo Puji Hospital, Ningbo, China; ^3^ Department of Pharmacy, The Seventh People's Hospital of Wenzhou, Wenzhou, China; ^4^ GCP certified site, Wuhan Jinyintan Hospital, Wuhan, China; ^5^ School of Pharmaceutical Sciences, Wenzhou Medical University, Wenzhou, China

**Keywords:** WZ51, myocardial fibrosis, 11β-HSD2, aldosterone, rat

## Abstract

Myocardial fibrosis (MF) is one of the leading causes of end-stage heart disease. Many studies have confirmed that inflammation caused by aldosterone may play an important role in the process of MF. A selective 11β-hydroxysteroid dehydrogenase type 2 (11β-HSD2) enzyme inhibitor can reduce the inactivation of cortisol, allowing cortisol to compete for mineralocorticoid receptors. This study investigated the protective effect of a novel selective 11βHSD2 inhibitor (WZ51) on MF and described its underlying mechanism. The administration of WZ51 in rats with MF significantly alleviated myocardial injury, accompanied by a decrease in lactate dehydrogenase and the creatine kinase myocardial band. Furthermore, WZ51 significantly inhibited the development of MF and increased the protein level of 11β-HSD2. The results of this study demonstrate that 11β-HSD2 plays an important pathological role in MF. Thus, WZ51 may be a potential therapeutic agent for the treatment of this condition.

## Introduction

Cardiovascular diseases, such as hypertension and myocardial infarction, are the leading cause of death worldwide (Sorriento and Iaccarino, 2019). Myocardial fibrosis (MF) can lead to myocardial remodeling, making it an important pathological feature of heart failure (Gulati et al., 2018; Kong et al., 2020). Slowing down the development of MF is an optimized strategy for reducing the risk of death from cardiovascular disease.

A large number of basic and clinical studies have shown that the pathogenesis of MF is closely related to the renin-angiotensin-aldosterone system (Brilla, 2000; Blauwet et al., 2009). The mineralocorticoid receptor (MR) is distributed in myocardial fibroblasts, cardiomyocytes, and vascular endothelial cells (Young, 2008). Myocardial hypertrophy, perivascular fibrosis, and myocardial interstitial fibrosis can be caused by aldosterone via the MR. At present, MR antagonists are the main drugs used for the clinical treatment of MF (Milliez et al., 2005). However, MR antagonists can cause adverse drug reactions such as hyperkalemia, gynecomastia, and erectile dysfunction in patients (Grune et al., 2016; Young and Adler, 2019; Khan et al., 2020).

It has been shown that cortisol and aldosterone are competitively bound to the MR (Funder and Myles, 1996; Chatterjee and Sikdar, 2014). The concentration and activity of cortisol are determined by the regulation of 11 β-hydroxysteroid dehydrogenase type 2 (11β-HSD2), a cortisol metabolic enzyme (Rengarajan and Balasubramanian, 2007). Several studies have demonstrated that the equilibrium concentrations of cortisol/cortisone in humans are homologous to corticosterone/11-dehydrocorticosterone in rodents. In other words, inhibition of 11β-HSD2 activity increases the plasma concentration of cortisol, which competitively antagonizes MR activation with aldosterone (De-An et al., 2010). WZ51, a derivative of curcumin, is a compound that strongly inhibits 11β-HSD2. Therefore, in this study, we investigated the effect of WZ51 on preventing MF.

## Materials and Methods

### Chemicals and Reagents

Deoxycortisol and MTT powder were purchased from Sigma-Aldrich Corporation. Spironolactone tablets were purchased from Hangzhou Minsheng Pharmaceutical Group Co., Ltd. GAPDH primary antibody was purchased from CST (Cell Signaling Technology. Lnc). CTGF and 11β-HSD primary antibodies were purchased from Abcam. All secondary antibodies were purchased from Shanghai Yeasen Biotechnologies Co., Ltd. Creatine kinase MB isoenzyme Assay Kit and Lactate dehydrogenase assay kit were purchased from Nanjing Jiancheng Bioengineering Institute. Masson’s Trichrome Stain Kit was purchased from Beijing Solarbio Science and Technology Co., Ltd.

### Animals

Male Wistar rats were treated with right nephrectomy at the initial stage of the experiment. Establishment of myocardial fibrosis model in rats: Right nephrectomy was performed after 10% chloral hydrate (400 mg/kg) intraperitoneal anesthesia. One week after the operation, drinking water (1% NaCl and 0.2% KCl) was given for 1 week. Deoxycorticosterone (aldosterone precursor, 60 mg/kg/d) was injected subcutaneously for 28 days. Sixty male Wistar rats were randomly divided into six groups (*n* = 10 per group): sham-operated control (Control) group, rats with right kidney surgically removed (Model) group, model rats treated with deoxycorticosterone (DOC, 60 mg/kg/d) group, DOC + spironolactone (SP, 20 mg/kg/d) group, DOC + WZ51−4 mg/kg/d group and DOC + WZ51−20 mg/kg/d group. The rats were injected subcutaneously for 28 consecutive days. After the intervention, the rats were sacrificed and serum and heart tissue were taken. Creatinine and serum lactate dehydrogenase were examined using the creatine kinase-myocardial band (CK-MB) kit and lactate dehydrogenase (LDH) kit. Histomorphology and collagen deposition were observed by hematoxylin and eosin staining and Masson staining of the rat heart tissue. All experimental procedures in present study were approved strictly by the Animal Care and Use Committee of Wenzhou Medical University.

### Western Blotting

The harvested heart tissues were homogenized and lysed, and the protein concentration determined. The cells were washed twice with ice-cold phosphate-buffered saline (PBS) and lysed in RIPA buffer. The lysates were centrifuged at 12,000 rpm for 10 min at 4°C. The protein concentrations were measured by the Bradford method. Protein samples were separated by 10% sodium dodecyl sulfate polyacrylamide gel electrophoresis and then electrotransferred to polyvinylidene difluoride membranes. After blocking in 5% milk for 2 h at room temperature, the membranes were incubated with primary antibodies overnight at 4°C. Membranes were washed three times with TBST (Tris-buffered saline, 0.1% Tween 20) for 5 min each and incubated with horseradish peroxidase-conjugated secondary antibody for 1.5 hat room temperature. Finally, the protein bands were visualized using a Gel Imaging System with an ECL Detection Kit. The Western blot bands were quantified by Image J analysis software, and values were normalized to their respective controls. Primary antibodies against 11β-HSD and connective tissue growth factor (CTGF) were purchased from Abcam (Cambridge, MA, United States), and antibodies against GAPDH were from Cell Signaling Technology (Danvers, MA, United States). Secondary antibodies were obtained from Santa Cruz Technology (Dallas, TX, United States).

### Immunohistochemistry

Hearts of mice were fixed in 4% (w/v) paraformaldehyde solution, and embedded. Each paraffin sample was sectioned into 5 μm thick, and selected hematoxylin and eosin (H&E), Sirius red (SR) and Masson as standard staining protocols. For immunohistochemical staining method, heart tissue sections were deparaffinized with xylene, rehydrated in gradient alcohol, subjected to antigen retrieval in citrate buffer solution (0.01 mol/L, pH 6.0) by microwaving, and then placed in a methanol solution of 3% hydrogen peroxide for 30 min at room temperature. Slides were blocked with PBS containing 1% BSA for 30 min, followed by incubating with primary antibody overnight at 4 °C (11β-HSD2, 1:500; CTGF, 1:1,000). Peroxidase-conjugated secondary antibodies were used as follows (Santa Cruz, 1:100 dilution; 1 h incubation). Finally, slides were counterstained with hematoxylin for 5 min, dehydrated, and mounted. Images were viewed and obtained under the electron microscope (Nikon, Japan). The gray value was calculated by ImageJ.

### Cells and MTT

H9C2 embryonic rat cardiomyocytes were cultured in Dulbecco’s Modified Eagle Medium (4.5 g/L) supplemented with 10% fetal bovine serum, penicillin (100 U/ml), and streptomycin (0.1 mg/ml). Cells were maintained at 37°C with 5% CO_2_ in air, and the medium was changed every 2–3 days. H9C2 cells were seeded in 96-well plates and incubated for 48 h with different drug concentrations after cell adherence. Then MTT was added and incubated for 4–6 h. After removing the culture, 150 μL dimethyl sulfoxide was added. The absorbance value of each hole at 490 nm was measured with an enzyme marker. Cell proliferation was indicated by the ratio of the photoabsorbance of the cell to that of the photochromic group. When incubated, cells would be pretreated with spironolactone or WZ51 or corticosterone for 30 min, then exposed to aldostersone (0.1 μmol/L).

### Statistical Analysis

Data are presented as the mean ± standard error of the mean. The statistical significance of differences between groups was obtained using the Student’s *t*-test or analysis of variance and multiple comparisons (GraphPad Pro8.0 software, San Diego, CA, United States).

## Results

### WZ51 Significantly Attenuates Myocardial Fibrosis in a rat Model

**FIGURE 1:**
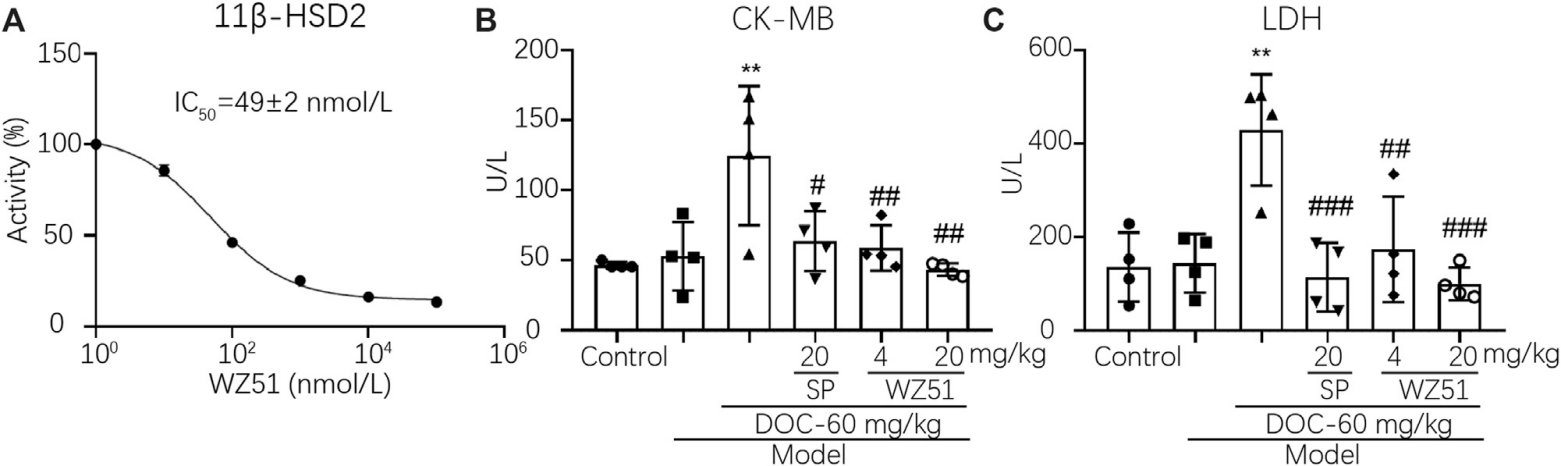
WZ51 significantly reduced myocardial injury in renal hypertensive rats. **(A)**. Inhibition of 11β-HSD2 by different concentrations of WZ51. **(B, C)**. Serum LDH and CK-MB of each group was determined using Creatine kinase MB isoenzyme Assay Kit and Lactate dehydrogenase assay kit following the recommended protocol (**p < 0.01 vs. CON; ^#^ *p* < 0.05, ^##^ *p* < 0.01, ^###^ *p* < 0.001 vs. DOC; *n* = 4). U/L = unit/L.

**FIGURE 2:**
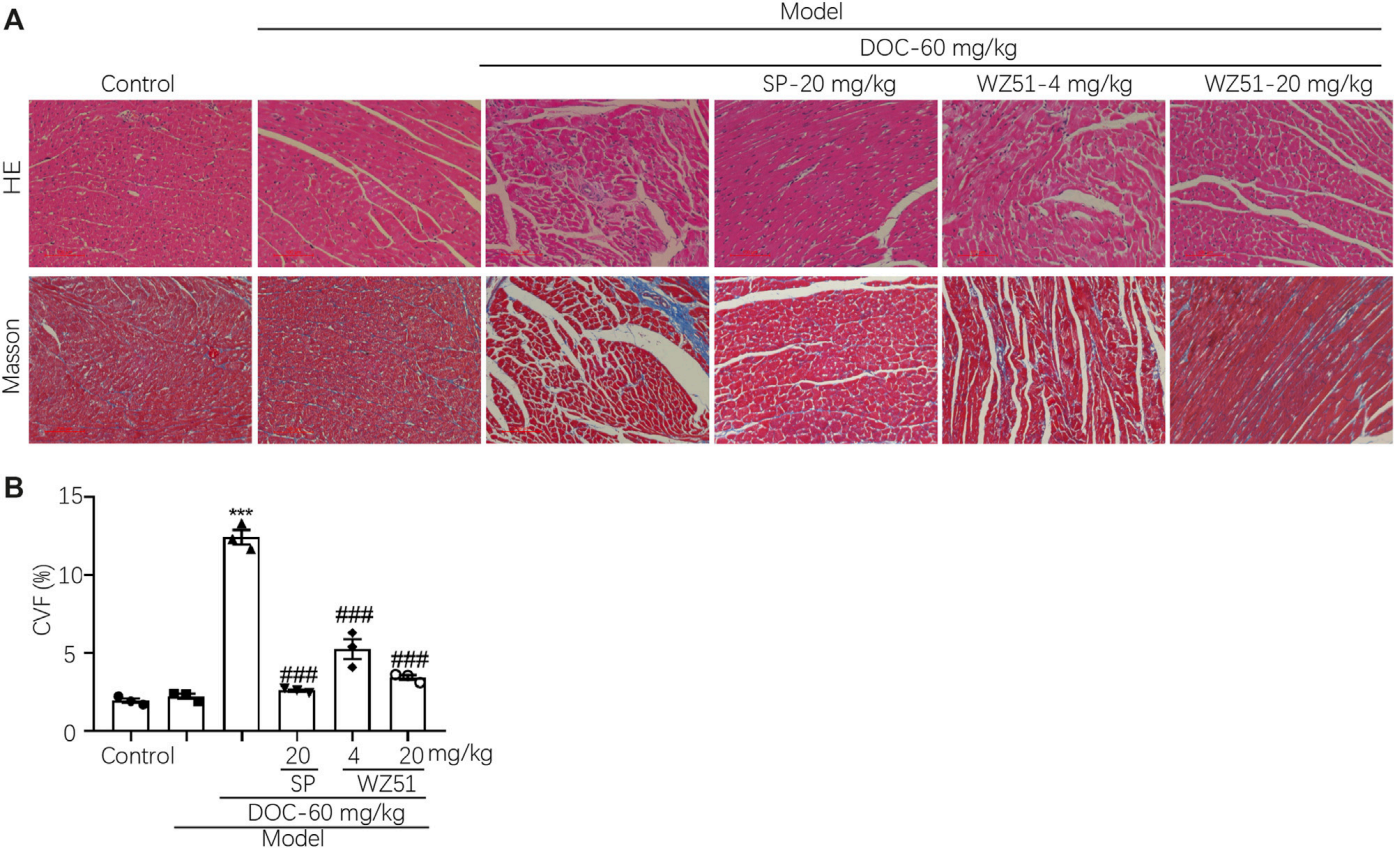
WZ51 attenuated MF in renal hypertensive rats. **(A, B)** After administration of WZ51 or SP, the heart tissues of each group were collected. Hematoxylin and eosin (HE) and Masson’s staining were performed. Representative images are shown (200 × magnification). **(C)** Statistical analysis of Masson’s staining (***p < 0.001 vs. CON; ^###^ *P* <0.0.001 vs. DOC; *n* = 0.3). CVF = collagen volume fraction.

Curcumin was discovered as a 11β-HSD inhibitor with higher potency than 11β-HSD1 (50% inhibitory concentration [IC_50_] = 2.30 μmol/L in humans, 5.80 μmol/L in rats) and 11β-HSD2 (IC_50_ = 14.63 μmol/L in humans, 11.90 μmol/L in rats). Therefore, our research team synthesized a lead compound named WZ51 based on the structure–activity relationship of curcumin. Interestingly, it was found to be a potent 11β-HSD2 inhibitor with a low IC_50_ (49 nmol/L) (Figure 1A). However, no obvious selective effect was observed as it also blocked the activity of 11β-HSD1 with an IC_50_ of 100 μmol/L (data not shown). Here, we investigated the effect of WZ51 on myocardial injury. To this end, renal hypertensive rats were established. In addition, deoxycorticosterone was injected subcutaneously to accelerate the process of MF by increasing the burden of the heart. LDH and CK-MB are important indicators of myocardial injury. In the rat model, LDH and CK-MB were significantly elevated in the DOC group (*p* < 0.01). However, WZ51 or SP administration markedly normalized these biomarkers (Figures 1B,C). Histologically, in the DOC group, the myocardial fiber gap widened and the myocardial transverse grain disappeared. Some cardiomyocytes died and those that survived were arranged in a loose and disordered manner. Nuclei of the cardiomyocytes contracted and dissolved. The MF degree in the SP group and WZ51–20 mg/kg group was significantly relieved compared with the DOC group. However, the difference between the WZ51−4 mg/kg group and DOC group was not obvious (Figure 2A). WZ51 treatment prevented the increased deposition of collagen and connective tissue in the heart when evaluated by Masson trichrome staining (Figure 2B).

### WZ51 Reduces the Expression of CTGF Accompanied by Upregulated Expression of 11β-HSD2

**FIGURE 3:**
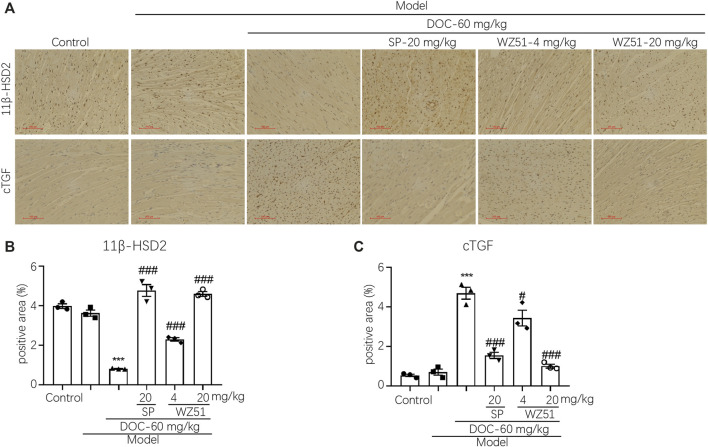
CTGF expression was upregulated, whereas 11β-HSD was inhibited in the heart tissues of renal hypertensive rats. **(A, B)** CTGF and 11β-HSD were determined by histochemistry. Representative images were shown (200 × magnification). **(C, D)** Statistical analysis of histochemistry (CTGF and 11β-HSD) (***p < 0.001 vs. CON; ^#^ *p* < 0.05, ^###^ *p* < 0.001 vs. DOC; *n* = 3).

Immunohistochemical detection of CTGF in the cardiac tissues of each group showed that WZ51 or SP prevented their increase in the heart tissues of renal hypertension rats (Figure 3A). This is consistent with the aforementioned histochemical data (Figures 2A,B). WZ51 played an effective role in preventing the development of MF in renal hypertensive rats. 11β-HSD2 is a cortisol metabolic enzyme that mediates the concentration and activity of cortisol. 11β-HSD2 histochemical detection results showed that the content of 11β-HSD2 in the DOC group was significantly reduced, which was reversed by WZ51 or SP treatment (Figure 3B).

### WZ51 Inhibits Aldosterone-Induced Fibrosis in H9C2 Cells

**FIGURE 4:**
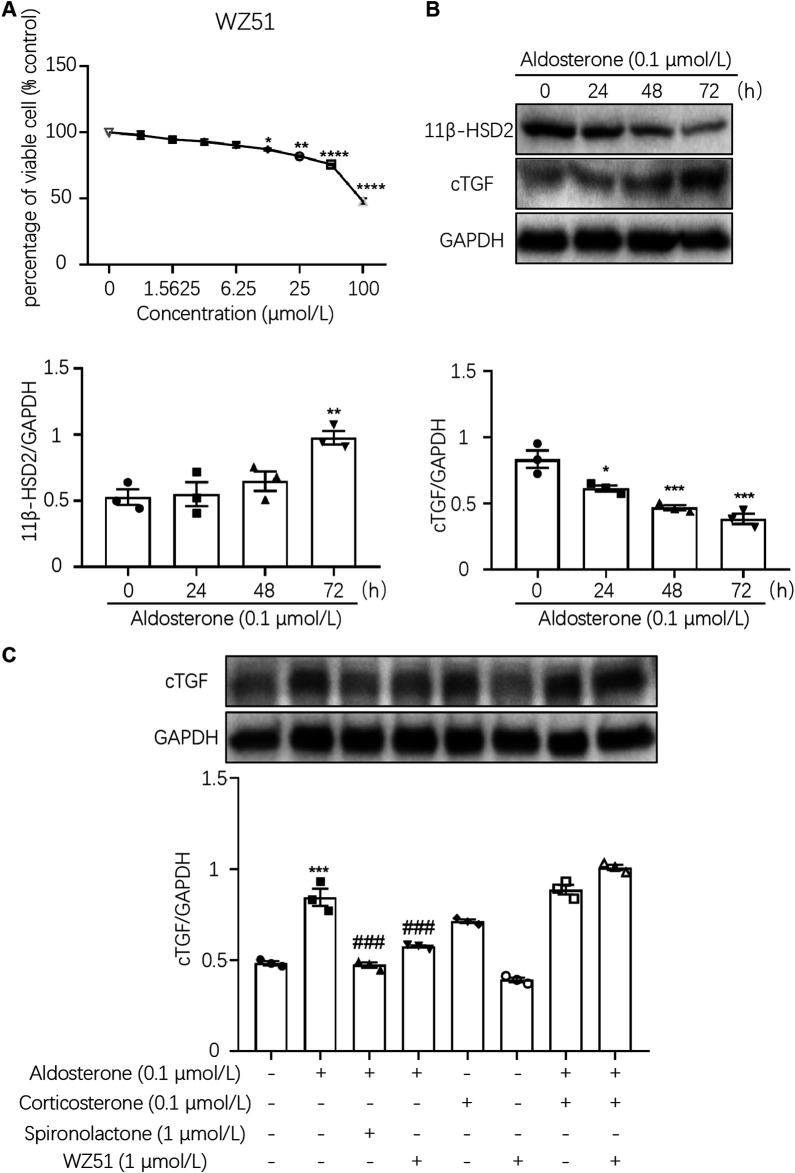
WZ51 mediated aldosterone-induced fibrosis in H9C2 cells. **(A)** Effect of WZ51 on the viability of H9C2 cells (72 h). Compared with the 0 μmol/L concentration group (*p < 0.05, **p < 0.01, ****p < 0.001, *n* = 3). **(B)** Changes in 11β-HSD2 and CTGF protein expression over time with aldosterone treatment (*p < 0.05, **p < 0.01, ***p < 0.001 vs. 0 h, *n* = 3). **(C)** Expression of CTGF was determined by Western blot analysis (***p < 0.001 vs. CON; ^#^ *p* < 0.05, ^###^ *p* < 0.001 vs. DOC; *n* = 3).

To determine the appropriate drug concentration, we conducted the MTT assay. The results showed that WZ51 inhibited the proliferation of H9C2 cells with an IC_50_ > 100 μmol/L. Therefore, 1.0 μmol/L was determined to be the drug concentration of WZ51 in follow-up cell experiments to ensure that there was no obvious inhibition of cell proliferation (Figure 4A). The expression of 11β-HSD2 decreased and that of CTGF increased with prolonged aldosterone treatment. The data indicated that the decrease in 11B-HSD2 expression was related to MF caused by aldosterone (Figure 4B). However, on aldosterone stimulation for 48 and 72 h, the expression of 11β-HSD2 and CTGF was significantly different (*p* < 0.01). Therefore, 48 h was determined as the drug action time for subsequent cell experiments. Consistent with animal experiment results, the results of Western blotting showed that WZ51 or SP administration reversed the aldosterone-induced increase in CTGF protein expression in H9C2 cells (Figure 4C).

## Discussion

With the global aging population, cardiovascular disease has become an important cause of disability and even death (Arnett et al., 2019). An important pathological feature of cardiovascular disease is MF, which can lead to ventricular remodeling (Hughes et al., 2019). MF is the abnormal deposition of extracellular matrix in the myocardium, which is characterized by increased collagen deposition in the interstitium, and an imbalanced and disordered collagen ratio (Essick and Sam, 2011).

In recent years, it has been found that aldosterone plays an important role in promoting MF (Brilla, 2000). It has the effect of promoting myocardial fibroblast proliferation and collagen deposition. In many studies, it has been confirmed that aldosterone antagonists can significantly reduce the occurrence of MF (Mizuno et al., 2001). Aldosterone-induced inflammation of myocardial tissue and blood vessels is characterized by the high expression of inflammatory factors before infiltration of mononuclear macrophages and fibrosis (Fuller et al., 2019). Inflammatory factors are key early features of MF induced by aldosterone-activated MRs (Young and Adler, 2019). In this study, after unilateral nephrectomy, rats were injected with exogenous DOC. It was observed that the activities of CK-MB and LDH in the serum of the DOC group were significantly increased, accompanied by severe myocardial interstitial fibrosis. After intervention with the aldosterone receptor antagonist SP, serum CK-MB, LDH activity, and MF indexes of rats in the SP group were significantly reduced. This indicates that exogenous aldosterone perfusion can induce MF, further confirming the important role of aldosterone in the process of inducing this condition.

Curcumin has cardiovascular protective effects such as antioxidation, free radical scavenging, anti-inflammation, antitumorigenic, and reversal of myocardial remodeling (Gupta et al., 2013; Zeng et al., 2015). WZ51 is a new drug derived from curcumin. It has been confirmed that it has no inhibitory effect on 11β-HSD1, but it can strongly inhibit 11β-HSD2. 11β-HSD is a cortisol-metabolizing enzyme. 11β-HSD has two isozymes: 11β-HSD1 and 11β-HSD2 (Chapman et al., 2013). 11β-HSD1 is an oxidoreductase that catalyzes the redox reaction between the ketone group at the C11 position and the hydroxyl group, converting inactive cortisone to active cortisol (Morris et al., 2003). 11β-HSD2 uses NAD + as a coenzyme to inactivate cortisol and corticosterone, making MR exclusively bind to aldosterone (Hofmann et al., 2001; De-An et al., 2010). The increase in cortisol concentration can compete with aldosterone to antagonize MR activation, thereby reducing aldosterone-induced MF (Young et al., 2007). In this study, rats treated with the curcumin analogue WZ51 had significantly lower serum CK-MB and LDH activity values; increased myocardial tissue 11β-HSD2 expression; and decreased CTGF expression, myocardial interstitial fibrosis, and blood vessels. The surrounding fibrosis was effectively relieved.

The mechanism of WZ51 against MF may be related to the selective inhibition of 11βHSD2 enzyme activity. WZ51 is capable of increasing the level of cortisol which compete for MR to form a cortisol-MR complex without transcription, and antagonize the combination of MR and mineralocorticoid aldosterone, thus blocking the damage of aldosterone to the myocardium, and finally alleviate the development of MF. In addition, As the activity of 11βHSD2 is inhibited, the body may produce corresponding feedback regulation, thus increasing 11βHSD2 protein.

## Data Availability

The original contributions presented in the study are included in the article/Supplementary Material, further inquiries can be directed to the corresponding authors.
